# Clinical response of vedolizumab at week 6 predicted endoscopic remission at week 24 in ulcerative colitis

**DOI:** 10.1002/jgh3.12630

**Published:** 2021-08-26

**Authors:** Daisuke Saito, Minoru Matsuura, Ryo Ozaki, Sotaro Tokunaga, Shintaro Minowa, Tatsuya Mitsui, Miki Miura, Akihito Sakuraba, Mari Hayashida, Jun Miyoshi, Tadakazu Hisamatsu

**Affiliations:** ^1^ Department of Gastroenterology and Hepatology Kyorin University School of Medicine Tokyo Japan

**Keywords:** colonoscopy, inflammatory bowel disease, ulcerative colitis

## Abstract

**Background and Aim:**

Vedolizumab is a humanized monoclonal antibody that selectively inhibits the migration of gut‐homing memory T cells into the intestinal submucosa by antagonizing the interaction of α_4_β_7_ integrin with MAdCAM‐1. Vedolizumab is employed for ulcerative colitis with moderate to severe activity; however, predictors of its clinical efficacy have not been established in real‐world clinical practice. We investigated the clinical characteristics predicting vedolizumab efficacy.

**Methods:**

This was a single‐center, retrospective, observational study that enrolled patients with ulcerative colitis at Kyorin University Hospital. Fifty‐two consecutive patients who started vedolizumab induction therapy and were tracked for minimum 14 weeks between August 2018 and February 2021 were included. Clinical and endoscopic disease activities were scored at baseline and at weeks 2, 6, and 14 with the Lichtiger index and at baseline and week 24 with the Mayo endoscopic subscore, respectively. Clinical remission, clinical response, and endoscopic remission were defined as Lichtiger index of ≤3, Lichtiger index of ≤10 with a reduction of minimum 3 points from baseline, and Mayo endoscopic subscore of ≤1, respectively.

**Results:**

In these cases, clinical response/remission rates at weeks 2, 6, and 14 were 26.9%/15.3%, 50.0%/46.3%, and 57.6%/50.0%, respectively. The endoscopic remission rate at week 24 was 60%. The clinical response at week 6 was significantly associated with endoscopic remission at week 24 after starting vedolizumab.

**Conclusions:**

In vedolizumab treatment for ulcerative colitis, the clinical response at week 6 can be a predictor for endoscopic remission at week 24.

## Graphical abstract

Clinical response of vedolizumab at week 6 predicted endoscopic remission at week 24 in ulcerative colitis with moderate to severe activity.

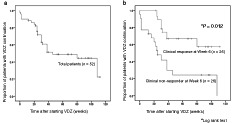

## Introduction

Ulcerative colitis (UC) is a chronic inflammatory disease of the colon that causes symptoms such as bloody stool, diarrhea, and abdominal pain.^1^, ^2^ The mechanisms involved in the development of UC remain to be fully elucidated, and its etiology remains unknown; however, genetic factors, intestinal microbiota, and environmental factors are believed to be involved in the development of UC.^3^, ^4^ The therapeutic strategy for UC has dramatically progressed recently, and various molecular targeting medications, including anti‐tumor necrosis factor‐α (TNF‐α) antibody, anti‐α_4_β_7_ antibody, anti‐IL12/23 p40 antibody, and Janus kinase inhibitor, are now used for UC treatment.

Vedolizumab (VDZ) is a humanized monoclonal antibody that selectively inhibits the migration of gut‐homing memory T cells into the gastrointestinal submucosa by antagonizing the interaction of α4β7 integrin with its ligand, MAdCAM‐1. GEMINI 1, a phase 3, randomized, placebo‐controlled trial, demonstrated that VDZ is effective for the induction and maintenance of clinical remission in patients with moderately to severely active UC with a favorable safety profile.^5^, ^6^ In Japan, a randomized, placebo‐controlled phase 3 study in 292 patients was performed.^7^, ^8^ The study showed that the clinical response rate at week 10 was higher in the VDZ group than in the placebo group (39.6% *vs* 32.9%) and the clinical remission rate at week 60 was significantly higher in the VDZ group than in the placebo group (56.1% *vs* 31.0%).

Currently, the therapeutic goal for UC is endoscopic remission or mucosal healing beyond the clinical response and remission.^9^, ^10^ The clinical symptoms do not always reflect the endoscopic findings,^11^ and some patients in clinical remission still have colonic mucosal inflammation.^12^ Several prospective studies have demonstrated that endoscopic remission is associated with reductions in disease relapse, hospitalizations, and surgery,^13^, ^14^, ^15^, ^16^, ^17^ as well as a lower cumulative risk of UC‐related dysplasia and colorectal cancer.^10^, ^18^ Recently, the Selecting Therapeutic Targets in Inflammatory Bowel Disease (STRIDE)^19^ and STRIDE II^20^ programs have suggested that a therapeutic target should be set to improve long‐term outcomes in inflammatory bowel disease treatment. Endoscopic remission is now widely accepted as a target in UC treatment because it is associated with a long‐term favorable prognosis. Conversely, no biomarkers or clinical properties that can predict endoscopic remission have been identified for any therapeutic option. In this molecular targeted therapy era, for the optimization of the clinical outcome as well as medical costs, predicting the effects of molecular targeted medications as early as possible is an unmet need. Here, we conducted a single‐center, retrospective cohort study to investigate the clinical properties that can be predictors of mid‐term endoscopic activity and practical goals in a real‐world clinical setting.

## Methods

### Patients

The clinical data of consecutive patients with UC who received VDZ as induction therapy and were followed at week 14 or later at Kyorin University Hospital between August 2018 and February 2021 were examined. UC diagnosis was made on the basis of clinical, endoscopic, radiological, and histological criteria.^1^, ^2^ The standard intravenous induction dose (300 mg) of VDZ was administered at weeks 0, 2, and 6, followed by maintenance therapy of an intravenous infusion every 8 weeks.

The data on the date of the first infusion of VDZ were collected as the baseline data: age, sex, disease duration, clinical disease activity, disease extent, previous anti‐TNF‐α antibody exposure, endoscopic activity (within 3 months before starting VDZ), concomitant treatment, and laboratory parameters, including C‐reactive protein (CRP), albumin, and hemoglobin.

### Clinical and endoscopic disease activity of UC

Clinical and endoscopic disease activities were assessed using the Lichtiger index (LI)^21^, ^22^ and Mayo endoscopic subscore (MES), respectively. Clinical remission and response were defined as LI of ≤3 and <10, with a reduction of minimum 3 points from the baseline score, respectively. Endoscopic remission was defined as MES of ≤1. Clinical disease activity was evaluated at baseline and at weeks 2, 6, and 14, and endoscopic disease activity was evaluated at baseline and at week 24 (or within 2 weeks before and after that). The patients who needed to withdraw VDZ owing to insufficient control of the disease activity before week 6 were defined as nonresponders at week 6.

### Statistical analysis

Statistical analysis was performed using SPSS software, version 25 (IBM Corp., Armonk, NY, USA). Categorical variables were analyzed using Fisher's exact test, and continuous variables were analyzed using Mann–Whitney *U* test. The cumulative administration continuation rate of VDZ was analyzed via the Kaplan–Meier method. Differences in the survival curves were assessed with the log‐rank test. *P* values of <0.05 were considered statistically significant.

### Ethical statement

This study was conducted in accordance with the guidelines of the Declaration of Helsinki and the approval of the Kyorin University Medical School Ethics Committee (approval number 687–01).

## Results

### Patients' characteristics

A total of 59 patients with UC were treated with VDZ between August 2018 and February 2021 at Kyorin University Hospital. Of these patients, 52 patients met the inclusion criteria of this study, whereas 7 patients were administered VDZ for the maintenance of remission. Demographic and clinical characteristics of patients at the start of treatment with VDZ are shown in Table 1. The most common extent of colitis was total colitis (42 patients, 80.8%). Among the 15 (28.8%) patients with a history of anti‐TNF‐α treatment, 4 patients and 1 patient were treated with 2 and 3 anti‐TNF‐α agents, respectively. As concomitant mediations, 5‐aminosalicylic acid, azathioprine, and prednisolone were used in 33 (63.4%), 14 (26.9%), and 13 (25.0%) patients, respectively. The median LI at baseline was 9 points (interquartile range [IQR] 7–10). Colonoscopy was performed within 3 months of the start of VDZ in 48 (92.3%) patients. Among the 48 patients, MES was 2 and 3 in 31 (64.5%) and 17 (35.4%) patients, respectively. No patients received additional treatment during the period from pretreatment colonoscopy to the start of VDZ. In terms of blood measurements, the median CRP, albumin, and hemoglobin levels were 0.44 (mg/dL), 3.70 (g/dL), and 12.5 (g/dL), respectively.

**Table 1:** Patients' characteristics at baseline (*n* = 52)

Characteristics	Total (*n* = 52)
Median age, year (IQR)	39 (23, 53)
Male, *n* (%)	36 (69.2)
Median body mass index, kg/m^2^ (IQR)	20.5 (17.8, 21.9)
Median disease duration, year (IQR)	5 (1.6, 8.3)
Extent of colitis, *n* (%)	
Pancolitis	42 (80.8)
Left‐sided colitis	10 (19.2)
Smoking status, *n* (%)	
Former smoker	6 (11.5)
Smoker	3 (5.8)
Nonsmoker	43 (82.7)
Prior anti‐TNF use, *n* (%)	
1	10 (19.2)
2	4 (7.7)
3	1 (1.9)
Concomitant drug, *n* (%)	
5‐aminosalicylic acid	33 (61.1)
Azathioprine	14 (26.9)
Prednisolone	13 (25.0)
Lichtiger index, median (IQR)	9 (7, 10)
Mayo endoscopic subscore, *n* (%)	
Mayo 2	31 (59.6)
Mayo 3	17 (32.7)
Median C‐reactive protein level, mg/dL (IQR)	0.44 (0.2, 1.8)
Median albumin, g/dL (IQR)	3.70 (3.2, 4.1)
Median hemoglobin, g/dL (IQR)	12.5 (10.7, 14.2)

IQR, interquartile range; TNF, tumor necrosis factor.

### Assessment of clinical and endoscopic disease activity

**Figure 1:**
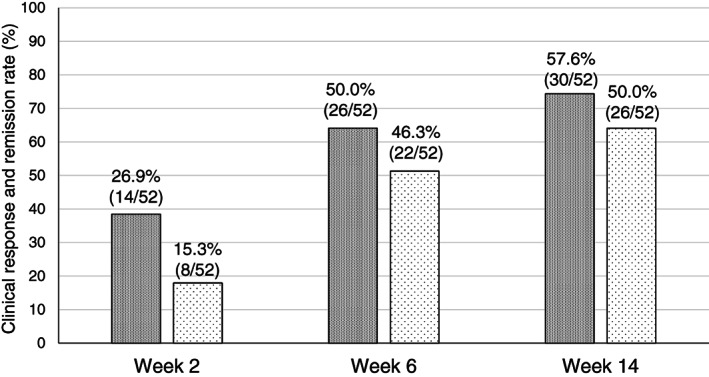
Clinical response and remission rates over time. We examined 52 patients who underwent induction therapy with vedolizumab; clinical response rates at weeks 2, 6, and 14 were 26.9% (14/52), 50.0% (26/52), and 57.6% (30/52), respectively, and clinical remission rates were 15.3% (8/52), 46.3% (22/52), and 50.0% (26/52), respectively. 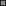, Clinical response; 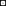, clinical remission.

The clinical response rate at weeks 2, 6, and 14 was 26.9% (14/52), 50.0% (26/52), and 57.6% (30/52), respectively. The clinical remission rate at weeks 2, 6, and 14 was 15.3% (8/52), 46.3% (22/52), and 50.0% (26/52), respectively. Both clinical response and remission rates markedly increased at week 6 compared with those at week 2 (Fig. 1). There was no significant difference between MES 2 *versus* 3 in the clinical response and remission at weeks 2, 6, and 14 (Table 2). The clinical response and remission rates at week 14 in patients naïve to anti‐TNF‐α agents (TNF‐naïve) and patients with a history of anti‐TNF‐α treatment (TNF failure) were 52.8% (19/36) and 43.7% (7/16), and there was no significant difference between the two groups (*P* = 0.764). Among the 52 patients, 35 underwent colonoscopy at week 24. The MES at week 24 was 3, 2, 1, and 0 in 7 (20.5%), 7 (20.5%), 8 (22.9%), and 13 (37.1%) patients, respectively (Fig. S1). The endoscopic remission rate at week 24 was 60.0% (21/35). All patients with endoscopic remission achieved steroid‐free clinical remission. The cumulative administration continuation rates of VDZ in those 35 patients after a colonoscopy at week 24 are shown in Figure S2. The cumulative administration continuation rate of patients who achieved endoscopic remission at week 24 was significantly higher (*P* = 0.002). This finding demonstrates that the medium‐term endoscopic remission at week 24 is associated with favorable long‐term outcomes in treatment with VDZ for UC.

**Table 2:** Comparison of clinical remission and response rate according to endoscopic activity at baseline

	MES 2 (*n* = 31)	MES 3 (*n* = 17)	*P* value^*^
Week 2			
Clinical response, *n* (%)	10 (32.2)	4 (23.5)	0.714
Clinical remission, *n* (%)	4 (12.9)	4 (23.5)	0.428
Week 6			
Clinical response, *n* (%)	14 (45.2)	12 (70.6)	0.132
Clinical remission, *n* (%)	13 (41.9)	9 (52.9)	0.551
Week 14			
Clinical response, *n* (%)	20 (64.5)	10 (58.8)	0.761
Clinical remission, *n* (%)	14 (45.2)	12 (70.6)	0.132

MES, Mayo endoscopic subscore.

^*^ Fisher's exact test.

### Predictors for medium‐term endoscopic remission with vedolizumab

Next, we assessed whether there were any clinical predictors for endoscopic remission at week 24. In the background information at baseline, there was no significant difference between patients who achieved endoscopic remission at week 24 (endoscopic remission group) and those who did not (non‐endoscopic remission group) (Table 3). In the comparisons of the clinical response/remission rate at weeks 2, 6, and 14, the rates at weeks 6 and 14 were significantly higher in the endoscopic remission group than in the non‐endoscopic remission group (each *P* < 0.001) (Table 4). Among the 24 patients with clinical response at week 6, 18 patients achieved endoscopic remission. The positive predictive value of clinical response at week 6 for endoscopic remission at week 24 was 0.75 (Table 5).

**Table 3:** Comparison of patient baseline characteristics between endoscopic remission and nonremission groups at week 24

	MES ≤1 (*n* = 21)	MES ≥2 (*n* = 14)	*P* value
Median age, year (IQR)	40 (25, 52)	37.0 (21.5, 48.5)	0.309^*^
Male, *n* (%)	16 (76.1)	10 (71.4)	0.752^*^
Median body mass index, kg/m^2^ (IQR)	20.8 (19.1, 21.9)	20.4 (18.2, 22.9)	0.624^*^
Median disease duration, year (IQR)	6.1 (3.1, 10.1)	5.0 (2, 7)	0.385^*^
Extent of colitis, *n* (%)			
Pancolitis	18 (85.7)	12 (85.7)	1.000^†^
Left‐sided colitis	3 (14.3)	2 (14.3)	
Prior anti‐TNF use, *n* (%)	7 (33.3)	9 (64.3)	0.093^†^
1	5 (23.8)	6 (42.9)	0.283^†^
2	1 (4.8)	3 (21.4)	0.279^†^
3	1 (4.8)	0 (0)	0.407^†^
Concomitant drug, *n* (%)			
5‐aminosalicylic acid	13 (61.9)	8 (57.1)	0.778^†^
Azathioprine	7 (33.3)	4 (28.6)	0.766^†^
Prednisolone	5 (23.8)	2 (14.2)	0.676^†^
Lichtiger index, median (IQR)	8 (7, 9)	8.5 (7.3, 10)	0.481^*^
Mayo endoscopic subscore, *n* (%)			
Mayo 2	14 (66.6)	9 (64.3)	0.884^†^
Mayo 3	4 (19.0)	5 (35.7)	0.432^†^
Median C‐reactive protein level, mg/dL (IQR)	0.32 (0.08, 1.88)	0.73 (0.37, 1.3)	0.151^*^
Median albumin, g/dL (IQR)	3.85 (3.4, 4.1)	3.5 (3.0, 3.8)	0.186^*^
Median hemoglobin, g/dL (IQR)	13.1 (11.1, 14.4)	12.1 (11.0, 13.7)	0.555^*^

IQR, interquartile range; TNF, tumor necrosis factor.

^*^ Mann–Whitney test.

^†^ Fisher's exact test.

**Table 4:** Comparison of clinical remission and response rate between the endoscopic remission and nonremission groups

	MES ≤1 (*n* = 21)	MES≥2 (*n* = 14)	*P* value^*^
Week 2			
Clinical response, *n* (%)	9 (42.9)	2 (14.3)	0.136
Clinical remission, *n* (%)	6 (28.5)	1 (7.1)	0.202
Week 6			
Clinical response, *n* (%)	18 (85.7)	6 (42.8)	0.007
Clinical remission, *n* (%)	17 (80.9)	2 (14.3)	<0.001
Week 14			
Clinical response, *n* (%)	21 (100.0)	2 (14.3)	<0.001
Clinical remission, *n* (%)	19 (90.4)	7 (50.0)	0.007

MES, Mayo endoscopic subscore.

^*^ Fisher's exact test.

**Table 5:** Association between clinical response at week 6 and endoscopic activity

		MES ≤1 at week 24	
		(+)	(−)
Clinical response at week 6	(+)	18	6
	(−)	3	8

Fisher's exact test. *P* = 0.007.

Sensitivity 0.85; Specificity 0.57; PPV 0.75; NPV 0.72.

MES, Mayo endoscopic subscore; NPV, negative predictive value; PPV, positive predictive value.

### Clinical response at week 6 predicts long‐term prognosis with vedolizumab

**Figure 2:**
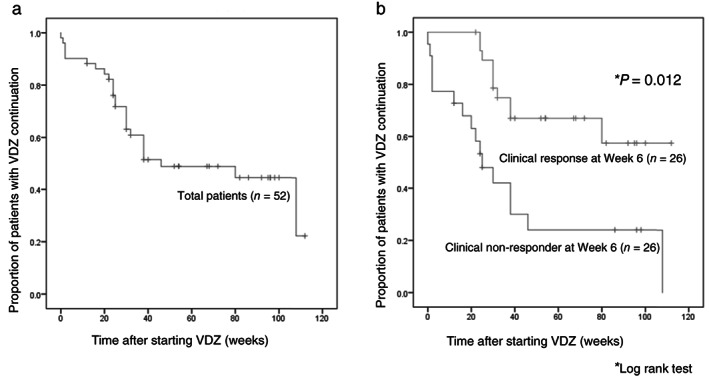
The cumulative vedolizumab (VDZ) administration continuation rate in all patients. (a) The cumulative administration continuation rate of VDZ in the 52 patients. (b) The administration continuation rate was significantly higher in the patients with clinical response at week 6 than in nonresponders (*P* = 0.012).

In this study, the median observation period was 38 weeks (range: 6–112, IQR: 24.5, 70). The cumulative administration continuation rate of VDZ in 52 patients is shown in Figure 2a. Among the 52 patients, VDZ was continued in 41 (78.8%) patients at week 24 and in 29 (55.8%) patients at week 48. Figure 2b presents the stratification with clinical response (*n* = 26) and nonresponse (*n* = 26) at week 6. The cumulative administration continuation rate of patients with clinical response at week 6 was significantly higher than that of the clinical nonresponders (*P* = 0.012).

## Discussion

Endoscopic remission is a crucial therapeutic goal in UC treatment. In the GEMINI 1 study, 51.6% of patients with UC treated with VDZ achieved MES of ≤1 at week 52.^5^, ^6^ Narula et al. reported that achievement of MES of ≤1 was noted in 29% and 62% patients at week 24 and week 48, respectively, in the VICTORY study.^23^ Several studies have assessed endoscopic remission with VDZ; however, the timing of colonoscopic evaluation and remission rate (30–60%) varied among the studies.^24^, ^25^, ^26^, ^27^ In our study, the endoscopic remission rate at week 24 was 60.0% (21/35), similar to a previous report evaluating real‐world outcomes.^27^ We observed that the cumulative VDZ administration continuation rate was significantly higher in patients who achieved endoscopic remission, and most of the patients in the non‐endoscopic remission group withdrew VDZ within the observation period. These findings suggest that endoscopic remission at week 24 is associated with a favorable long‐term prognosis with treatment using VDZ and is a treatment target for patients with UC treated with VDZ.

Meanwhile, it is clinically important to predict the endoscopic remission at week 24 as early as possible after starting VDZ to optimize the therapeutic strategy for each patient. In this study, we demonstrated that the clinical response at week 6 was associated with endoscopic remission at week 24. Its positive predictive value and negative predictive value for endoscopic remission at week 24 of the clinical response at week 6 were 75.0% and 72.0%, respectively. In addition, the continuation rate of VDZ was significantly higher in patients with clinical response at week 6 than in those with nonresponders. These findings showed that an early clinical response to VDZ (week 6) can be a favorable predictor of treatment with VDZ for UC. Also, in our study, endoscopic remission rate in clinical nonresponders at week 6 was significantly lower, and the evaluation as a predictor was equivalent to the endoscopic remission rate in clinical responders at week 6. From this, clinical nonresponders at week 6 may predict subsequent unsuccessful treatment of VDZ. That is, in VDZ treatment, we should consider enhancement treatment for UC depending on clinical response at week 6. Nagahori et al. reported that early symptomatic improvement predicted the treatment response at week 10 in TNF‐naïve patients.^28^ Furthermore, Bertanira et al. showed that mucosal healing at week 54 was associated with higher interleukin (IL)‐8 values at baseline and with a significant reduction in IL‐6 and IL‐8 levels over the first 6 weeks.^29^ Cumulatively, although various factors have been proposed, the continuation of VDZ might need careful consideration, particularly in patients without early clinical response.

Conversely, among patients who showed a clinical response at week 6, there were 6 patients with loss of response (LOR) to VDZ, and these patients withdrew VDZ. In GEMINI1,^5^, ^6^ the positive rate of anti‐VDZ antibody was 6% (39/620) and the positive rate of neutralizing antibody was 4% (27/620). In a study on LOR of VDZ, Shmidt et al. reported that cumulative rates for LOR of VDZ in patients with UC were 15% at 6 months and 30% at 12 months.^30^ It has been reported that VDZ infusion interval shortening^29^ and dose escalation^31^ are effective in patients with UC who present with LOR during treatment with VDZ. In Japan, however, these treatment enhancements have not been approved for VDZ. Further studies on the impact of LOR on long‐term prognosis and the measures against LOR in treatment with VDZ for UC are warranted.

Our study has several limitations. First, this was a single‐center, retrospective study with a limited number of cases. Second, there could be a selection bias in participants, considering that most VDZ administrations were decided by physicians in each outpatient clinic. Third, we cannot exclude the possibility of interobserver bias in the endoscopic assessment because this study did not employ the central evaluation system. Fourth, owing to the small number of cases, the correlation between TNF‐naïve/failure and endoscopic remission could not be analyzed. Nonetheless, we believe that our study, based on real‐world clinical data, provides significant insights into the clinical efficacy of VDZ and optimization of treatment with VDZ for UC.

## Conclusion

We examined 52 patients with UC who were treated with VDZ remission‐induction therapy. The clinical response rate at week 6 was 50.0% (26/52). The endoscopic remission rate at week 24 was 60% (21/35) and was associated with the clinical response at week 6.

## Supporting information

**Figure S1.** Endoscopic findings at baseline and week 24. Of the 52 patients, 35 underwent colonoscopy at week 24. The MES at week 24 was of 3 (20.5%) in 7 cases, 2 (20.5%) in 7 cases, 1 (22.9%) in 9 cases, and 0 (37.1%) in 13 cases.

**Figure S2.** Cumulative VDZ administration continuation rate after colonoscopy at week 24. The cumulative administration continuation rate of VDZ after colonoscopy at week 24 was analyzed in 35 patients. Patients with endoscopic remission showed a better continuation rate than those without endoscopic remission (*p* = 0.002).
