# Supplementary material for: Clinical response of vedolizumab at week 6 predicted endoscopic remission at week 24 in ulcerative colitis
Source: JGH Open. 2021 Aug 26;5(9):1056–62. doi: 10.1002/jgh3.12630 (PMC8454470; doi:10.1002/jgh3.12630)
Supplement: Supplementary file 1 — Figure S1. Endoscopic findings at baseline and week 24. Of the 52 patients, 35 underwent colonoscopy at week 24. The MES at week 24 was of 3 (20.5%) in 7 cases, 2 (20.5%) in 7 cases, 1 (22.9%) in 9 cases, and 0 (37.1%) in 13 cases. [file JGH3-5-1056-s002.pptx]

## Slide 1
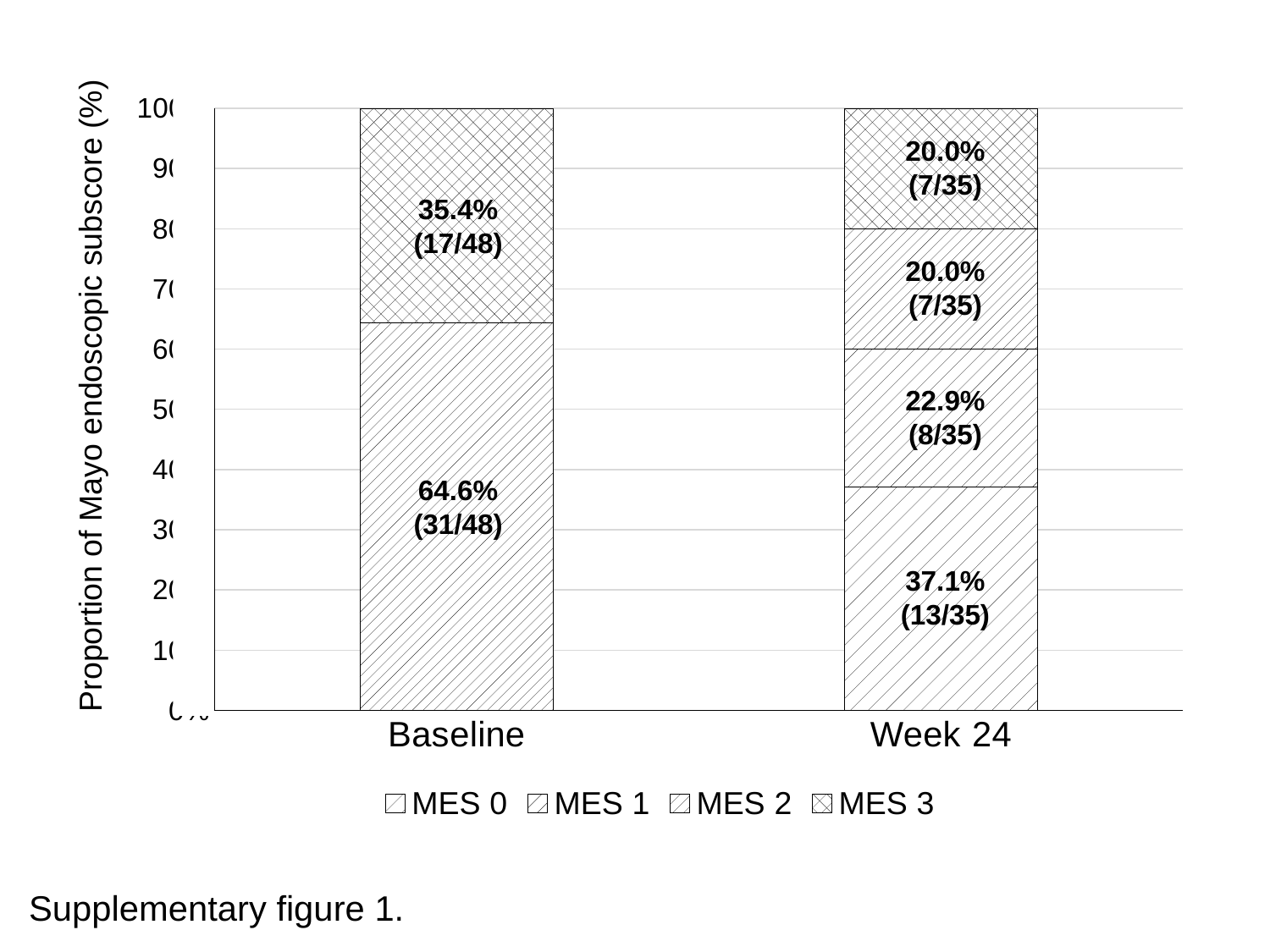

### Chart
| Category | MES 0 | MES 1 | MES 2 | MES 3 |
|---|---|---|---|---|
| Baseline | 0.0 | 0.0 | 64.4 | 35.6 |
| Week 24 | 37.1 | 22.9 | 20.0 | 20.0 |20.0%
(7/35)
35.4%
(17/48)
20.0%
(7/35)
Proportion of Mayo endoscopic subscore (%)
22.9%
(8/35)
64.6%
(31/48)
37.1%
(13/35)
Supplementary figure 1.
