# Supplementary material for: Clinical response of vedolizumab at week 6 predicted endoscopic remission at week 24 in ulcerative colitis
Source: JGH Open. 2021 Aug 26;5(9):1056–62. doi: 10.1002/jgh3.12630 (PMC8454470; doi:10.1002/jgh3.12630)
Supplement: Supplementary file 2 — Figure S2. Cumulative VDZ administration continuation rate after colonoscopy at week 24. The cumulative administration continuation rate of VDZ after colonoscopy at week 24 was analyzed in 35 patients. Patients with endoscopic remission showed a better continuation rate than those without endoscopic remission (p = 0.002). [file JGH3-5-1056-s001.pptx]

## Slide 1
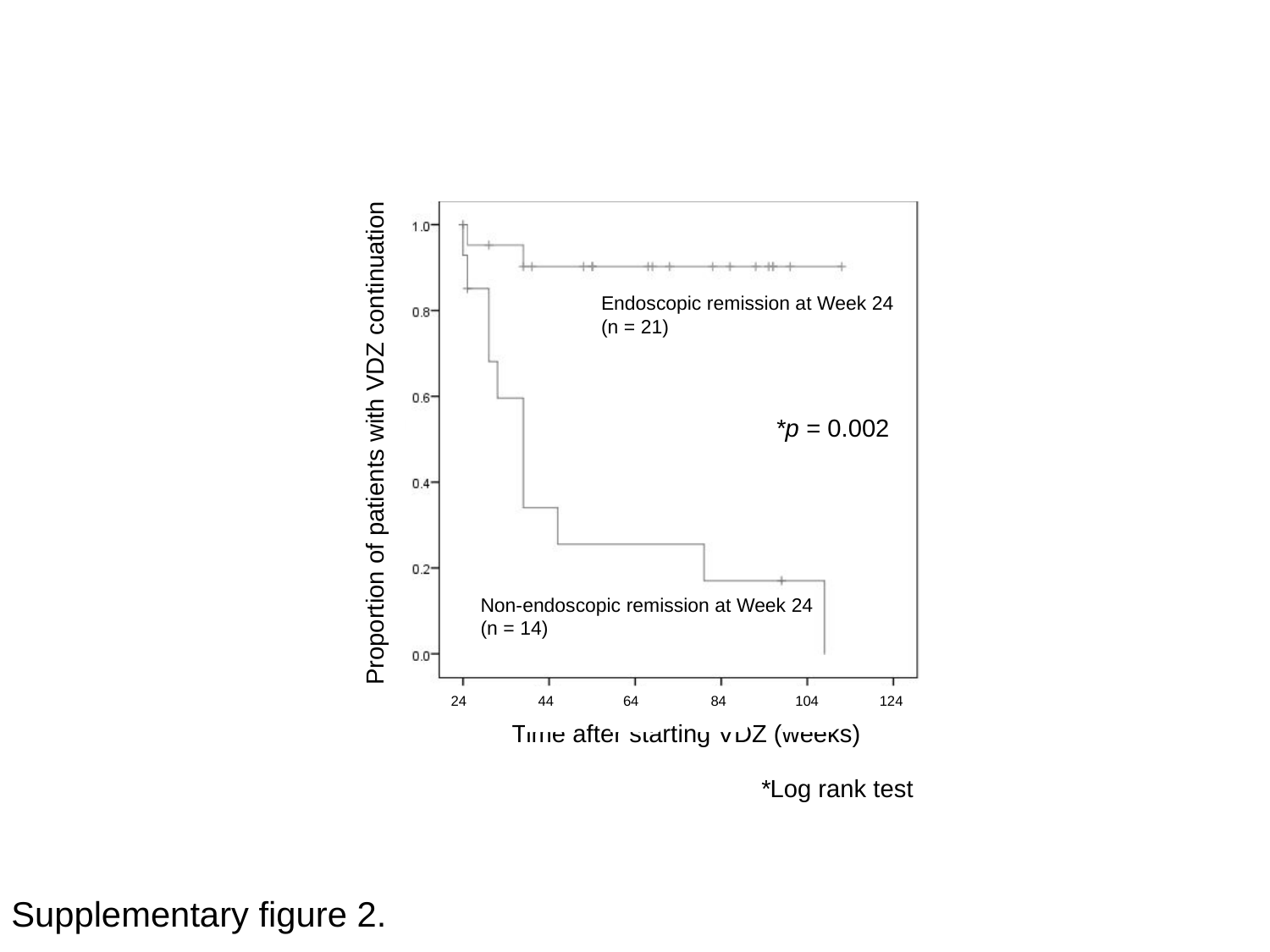

Endoscopic remission at Week 24
(n = 21)
*p = 0.002
Proportion of patients with VDZ continuation
Non-endoscopic remission at Week 24
(n = 14)
24
44
64
84
104
124
Time after starting VDZ (weeks)
*Log rank test
Supplementary figure 2.
